# Histological Validation of measurement of diffuse interstitial myocardial fibrosis by myocardial extravascular volume fraction from Modified Look-Locker imaging (MOLLI) T1 mapping at 3 T

**DOI:** 10.1186/s12968-015-0150-0

**Published:** 2015-06-11

**Authors:** Christophe de Meester de Ravenstein, Caroline Bouzin, Siham Lazam, Jamila Boulif, Mihaela Amzulescu, Julie Melchior, Agnès Pasquet, David Vancraeynest, Anne-Catherine Pouleur, Jean-Louis J. Vanoverschelde, Bernhard L. Gerber

**Affiliations:** Division of Cardiology, Department of Cardiovascular Diseases, Cliniques Universitaires St. Luc UCL, Av Hippocrate 10 / 2806, B-1200 Woluwe St. Lambert, Belgium; Pôle de Recherche Cardiovasculaire (CARD), Institut de Recherche Expérimentale et Clinique (IREC), Université Catholique de Louvain, Brussels, Belgium

## Abstract

**Background:**

Gadolinium (Gd) Extracellular volume fraction (ECV) by Cardiovascular Magnetic Resonance (CMR) has been proposed as a non-invasive method for assessment of diffuse myocardial fibrosis. Yet only few studies used 3 T CMR to measure ECV, and the accuracy of ECV measurements at 3 T has not been established. Therefore the aims of the present study were to validate measurement of ECV by MOLLI T1 mapping by 3 T CMR against fibrosis measured by histopathology. We also evaluated the recently proposed hypothesis that native-T1 mapping without contrast injection would be sufficient to detect fibrosis.

**Methods:**

31 patients (age = 58 ± 17 years, 77 % men) with either severe aortic stenosis (*n* = 12) severe aortic regurgitation (*n* = 9) or severe mitral regurgitation (*n* = 10), all free of coronary artery disease, underwent 3 T-CMR with late gadolinium enhancement (LGE) and pre- and post-contrast MOLLI T1 mapping and ECV computation, prior to valve surgery. LV biopsies were performed at the time of surgery, a median 13 [1–30] days later, and stained with picrosirius red. Pre-, and post-contrast T1 values, ECV, and amount of LGE were compared against magnitude of fibrosis by histopathology by Pearson correlation coefficients.

**Results:**

The average amount of interstitial fibrosis by picrosirius red staining in biopsy samples was 6.1 ± 4.3 %. ECV computed from pre-post contrast MOLLI T1 time changes was 28.9 ± 5.5 %, and correlated (r = 0.78, p < 0.001) strongly with the magnitude of histological fibrosis. By opposition, neither amount of LGE (r = 0.17, *p* = 0.36) nor native pre-contrast myocardial T1 time (r = −0.18, *p* = 0.32) correlated with fibrosis by histopathology.

**Conclusions:**

ECV determined by 3 T CMR T1 MOLLI images closely correlates with histologically determined diffuse interstitial fibrosis, providing a non-invasive estimation for quantification of interstitial fibrosis in patients with valve diseases. By opposition, neither non-contrast T1 times nor the amount of LGE were indicative of the magnitude of diffuse interstitial fibrosis measured by histopathology.

## Background

Measurement of extracellular volume (ECV) by Gd-DTPA Cardiovascular magnetic resonance (CMR) has recently been proposed as a non-invasive method to quantify myocardial interstitial volume and fibrosis [1, 2]. Several studies have compared CMR ECV estimated by different approaches [3–7] against fibrosis by histopathology, yet all these studies were performed only at 1.5 T. Presently, 3 T systems are more and more commonly used for cardiac MR. Yet the accuracy of measurement of ECV at 3 T has not yet been evaluated. Therefore the aims of the present study were to validate measurement of ECV derived from MOLLI T1 mapping at 3 T vs histopathology. An additional aim of our study was to examine the recently proposed hypothesis that native-non contrast enhanded-T1 mapping would be sufficient to detect myocardial fibrosis [8–10]. Finally we also sought to evaluate the relation between severity of late Gd-enhancement (LGE) and histological fibrosis.

We thus studied 31 patients scheduled to undergo aortic or mitral valve surgery by 3 T CMR and compared ECV, LGE and pre and post contrast T1 mapping against measurements of myocardial fibrosis by histopathology.

## Methods

### Study protocol and population

Consecutive patients scheduled to undergo surgery for either severe aortic stenosis, severe aortic or severe mitral regurgitation at our institution (Comité éthique hosptalo-facultaire des Cliniques Universitaires St. Luc, Brussels, Belgium), were considered eligible for inclusion in the study. Recruitment was performed between May 2013 and May 2014. Exclusion criteria were: history of prior myocardial infarction; presence of significant coronary artery disease on preoperative angiography; and contraindications to CMR (ferrometallic cerebral aneurysm clips, pacemaker or implantable defibrillator, or severe claustrophobia), or to injection of Gd based contest agent (ie allergy to contrast media or renal insufficiency with GFR-MDRD < 30 ml/min/1.73 m2). The study protocol consisted of a Gd-enhanced MR prior to cardiac surgery followed by myocardial biopsy at the time of surgery. The protocol was IRB approved and patients were included after giving written informed consent to participating in this study. 31 participants fulfilled inclusion criteria and consented to participating in the study (Table 1). Scan-rescan reproducibility of pre-, post- T1 and ECV was performed in 11 other subjects: 5 healthy volunteers (3 males 44 ± 11 years), 4 patients with aortic stenosis (3 males, mean age 60 ± 20 years) and 2 patients with mitral regurgitation (2 male, mean age 65 ± 7 years).

**Table 1:** Patients characteristics

	All patients	Aortic stenosis	Aortic regurgitation	Mitral regurgitation
	(*n* = 31)	(*n* = 12)	(*n* = 9)	(*n* = 10)
Age (year)	58 ± 17	70 ± 12	47 ± 17	54 ± 14
Male Gender (n, %)	24 (77 %)	10 (83 %)	7 (78 %)	7 (70 %)
Weight (kg)	79 ± 17	75 ± 17	88 ± 18	74 ± 12
Height (cm)	174 ± 10	171 ± 8	175 ± 10	176 ± 11
BMI (kg/m2)	26 ± 5	26 ± 5	29 ± 4	24 ± 3
Hypertension (n, %)	15 (48 %)	9 (75 %)	5 (56 %)	1 (10 %)
Smoking (n, %)	7 (23 %)	3 (25 %)	3 (33 %)	1 (10 %)
Hyperlipemia (n, %)	8 (26 %)	8 (67 %)	0 (0 %)	0 (0 %)
Diabetes Mellitus (n, %)	2 (6 %)	2 (17 %)	0 (0 %)	0 (0 %)
NYHA Class I/II	15 (48 %) / 16 (52 %)	3 (25 %) / 9 (75 %)	7 (78 %) / 2 (22 %)	5 (50 %) / 5 (50 %)
Atrial fibrillation (%)	5 (16 %)	4 (33 %)	0 (0 %)	1 (10 %)
Heart rate (bpm)	65 ± 7	65 ± 6	68 ± 8	64 ± 8
Systolic Blood Pressure (mmHg)	134 ± 18	134 ± 11	152 ± 19	119 ± 10
Diastolic Blood Pressure (mmHg)	72 ± 13	71 ± 12	71 ± 19	74 ± 9

BMI: Body Mass Index, NYHA: New York Heart Association SD: Standard Deviation

### Cardiac MR

Cardiac MR was performed using a 3 Tesla system (Ingenia, Philips Medical Systems, Best, the Netherlands). To assess LV myocardial function and mass, 10 to 12 consecutive short-axis images and 2-, 3- and 4-chamber long axis image of the LV were acquired using a cine steady state free precession sequence. Then, mid-ventricular short axis Modified look locker (MOLLI) images were acquired for T1 determination using an 11 image, 18 heart-beat 3-(3)-3-(3)-5 SSFP sequence. Imaging parameters were: Field of view: 340 mm, slice thickness 8 mm, TR: 2.6 ms TE 1.03 ms, matrix 172 × 150 pixels resulting in a resolution of 2x2.6 mm, Sense Factor 2, trigger delay end-diastole, inversion times ranging from 150–3287 ms. Then, a total dose of 0.2 mmol/kg gadobutrol (Gadovist, Schering) was injected in a 2 phase protocol: 3 mL gadobutrol were infused as bolus pushed by a 15 mL saline at 3 mL/s, 15 s later, the remaining contrast dose followed by 20 mL saline were infused at a slower rate of 1 mL/s. Ten to 15 min after contrast injection, short- and long-axis 2D inversion recovery LGE images were acquired with an inversion-recovery gradient-echo imaging sequence to evaluate focal myocardial fibrosis, as previously described [11]. Finally, post-contrast (15 min post contrast) MOLLI T1 mapping was repeated in identical prescription as pre-contrast T1 mapping.

For scan-rescan reproducibility, after acquisition of the first baseline T1 mapping, the patient was removed from the scanner and asked to stand up, then was put back in the scanner for the acquisition of the second set of images. A different scanner operator, starting from new scout images, acquired images for the second scan. After contrast administration, the same procedure was performed for post-contrast T1 maps. Post-contrast images were repeated without additional contrast agent administration between both scans.

### CMR data analysis

CMR images were anonymized and analyzed in double by two experienced (CD with 6 years of CMR experience, MA with 4 year’s CMR experience level and 3 Euro-CMR certification) observers blinded to clinical data.

*Pre and post-contrast MOLLI images* were processed using the open-source software MRmap v1.4 [12] under IDL. Images were corrected for respiratory motion when needed, and T1 maps were generated by fitting pixels to the equation s(t) = a – b exp (t/T1*), and T1 = T1*((b/a-1), where a and b are constants, t is time and s(t) signal intensity at time t. The generated pre- and post-contrast T1 maps were stored in DICOM format and imported into Osirix software (Pixmeo; Switzerland; version 5.8). Pre and post contrast blood T1 times were measured on a region of interest manually drawn in the center of the blood pool. Pre and post myocardial T1 times were measured in a segment corresponding approximately  to the site of the biopsy (depending on the patient either the anterior or antero-septal wall). To evaluate the influence of segmental heterogeneity on measurements we also measured T1 times, in 6 different regions of the myocardium (anterior-, anterolateral, inferolateral, inferior, inferoseptal, anterosepal) and one global region of interest encompassing the entire left ventricular wall. The partition coefficient lambda (λ) and ECV were computed as: 

$$ \lambda =\frac{1/T1 myocardium\kern0.5em  postC-1/T1 myocardium\kern0.5em  preC}{1/T1 blood\kern0.5em  postC-1/T1 blood\kern0.5em  preC} $$

*ECV=λ (1-Hematocrit). Late enhanced CMR* short axis images were analyzed using the freely available software software Segment v1.9 (http://www.medviso.com/products/segment) with a fully automated method [13] validated in an animal model of experimental infarction. The method automatically computes mean and standard deviation of signal intensity in 5 sectors per slice. The region with the lowest mean signal intensity is considered ‘remote’ myocardium. LGE regions are considered > 2.4 SD of remote after correction for partial volume effects. Isolated LGE regions < 1.5 ml, unless being >1 % of LV volume or being the largest LGE area in the image volume were deducted. The pattern of LGE was assessed by 2 independent observers (BG- 15 years of CMR experience, and MA 4 years CMR experience, both EuroCMR level 3 certified) who were blinded to the clinical and histopathological data. Discordant findings were resolved by consensus.

### Left ventricular biopsy

In each patient, 2–4 myocardial biopsy samples weighing approximately 25–75 mg were gathered, of which the largest sample was analysed for histopathology, and the remaining were preserved for other use. In patients undergoing open-chest aortic valve replacement for aortic valve stenosis, or aortic valve repair for aortic regurgitation, biopsies were sampled for full width of myocardium, under direct vision by means of a Tru-Cut® biopsy needle (CareFusion, Waukegan, IL) in the anterior wall. Patients undergoing mitral valve repair for mitral valve regurgitation by either Port-Access or Da-Vinci minimal-access had samples taken under endoscopic vision taken by a surgical scissor from the anterior septum. Samples were immediately fixed in 10 % buffered formalin, embedded in paraffin, sectioned, and stained with picrosirius red. Stained sections were digitalized with a SCN400 slide scanner (Leica Biosystems, Wetzlar, Germany). Quantification was performed using TissueIA software (Leica Biosystems, Dublin, Ireland). After elimination of artifacts and perivascular fibrosis, area occupied by interstitial fibrosis was expressed a percentage of total endomyocardial area. Four different histological slices were analyzed per patient and the average of the quantification of the different specimens was considered the final value of fibrosis for the patient.

### Statistical methods

Statistical analyses were performed using SPSS version 20.0 software (IBM Inc, Chicago, IL). Continuous variables were expressed as mean ± one standard deviation (SD) or medians [quartiles] if not normally distributed; categorical variables were reported as counts and percentages. All tests were 2 sided and p value < 0.05 was considered statistically significant.

To compare baseline characteristics, T1 times and ECV of patients with different type of valve disease, ANOVA or Chi square tests were used. The Pearson correlation coefficient was employed to examine the relationship between percent LGE, pre and post contrast T1 times and ECV by CMR versus quantitative interstitial fibrosis by histopathology. Intra and inter observer and scan-rescan reproducibility of T1 times and ECV and histopatholoy were assessed with Bland-Altman methods and intraclass correlation coefficient.

## Results

### Patient population

**Fig. 1:**
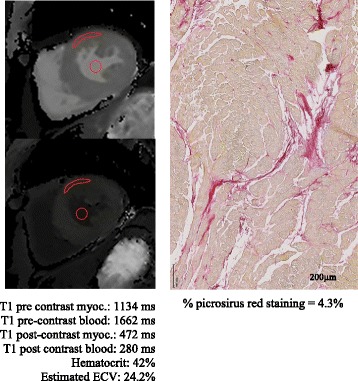
Example of pre and post contrast T1 maps and histophatology. Pre and post contrast T1 Maps s and fitted T1 values (left panel) and c corresponding histopathology (right panel)

The characteristics of the study population are presented in Table 1. 12 patients had severe aortic stenosis, 10 severe mitral regurgitation, 9 severe aortic regurgitation. Patients with aortic stenosis were significantly older (mean age 70 ± 12 years, p = 0.002) than patients with mitral regurgitation (54 ± 14 years) or aortic regurgitation (47 ± 17 years). The median delay between CMR and surgery/biopsy was 13 days [1-29] days. Figure 1 illustrates CMR T1 mapping and histopathology of a typical patient.

### Myocardial fibrosis by histopathology

Myocardial biopsy was collected without complication in all subjects. The average percentage of interstitial fibrosis by histopathology was 6.1 ± 4.3 %, and ranged from 2.3 % to 21.0 %. The amount of interstitial fibrosis was higher in patients with aortic stenosis (7.3 ± 5.4 % [2.4;21.0]) than in patients with aortic (5.2 ± 2.5 % [2.8;9.4]) and mitral regurgitation (5.4 ± 4.1 % [2.3;15.4]), however our study did not have sufficient power (β = 0.8) to demonstrate statistical significance (*p* = 0.46 by ANOVA) for this difference.

### CMR-LGE and its relation to histology

**Fig. 2:**
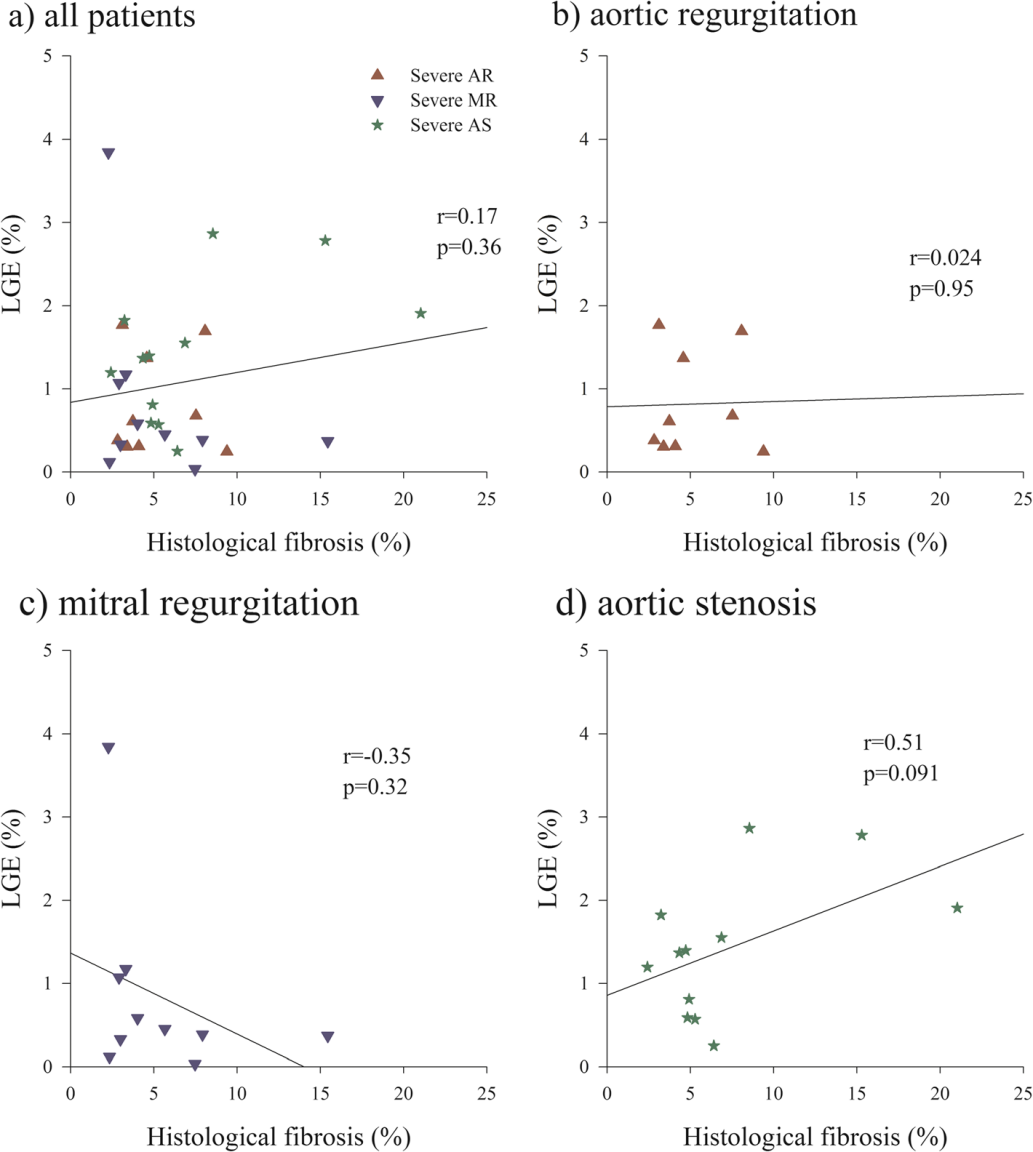
Correlation between quantitatively measured LGE and fibrosis by histopathology. Correlation between quantitatively measured LGE and % histological fibrosis for the whole population panel (**a**) and individual curves in patients with aortic regurgitation panel (**b**) mitral regurgitation panel (**c**) and aortic stenosis panel (**d**)

17 patients presented small areas of LGE in non-infarct patterns: ie focal lines (septal stripe) in 1 patient, and focal spots or diffuse areas of LGE in midventricular or epicardial location in 16 patients. No patient had sub-endocardial or transmural LGE. Also no patient had LGE in the areas used for T1 quantification. The average percentage of myocardium affected by LGE measured by the automatic quantification software was 1.1 ± 0.9 % [0.0;3.8] and did not significantly differ (*p* = 0.20) between patients with aortic stenosis (1.4 ± 0.8 %, [0.3;2.9]), aortic regurgitation (0.8 ± 0.6 % [0.24;1.77]) or mitral regurgitation (0.84 ± 1.12 % [0.03;3.84]). There was no significant relationship between the amount of LGE and the magnitude of diffuse fibrosis determined by histology in either the whole group of subjects (r = 0.17, p = 0.36 Fig. 2) or in patients with severe AR, (r = 0.024, *p* = 0.95), severe MR (r = −0.35, *p* = 0.32), or severe AR (r = 0.51, *p* = 0.091) alone.

### T1 times before and after contrast and ECV and its relation to histology

**Fig. 3:**
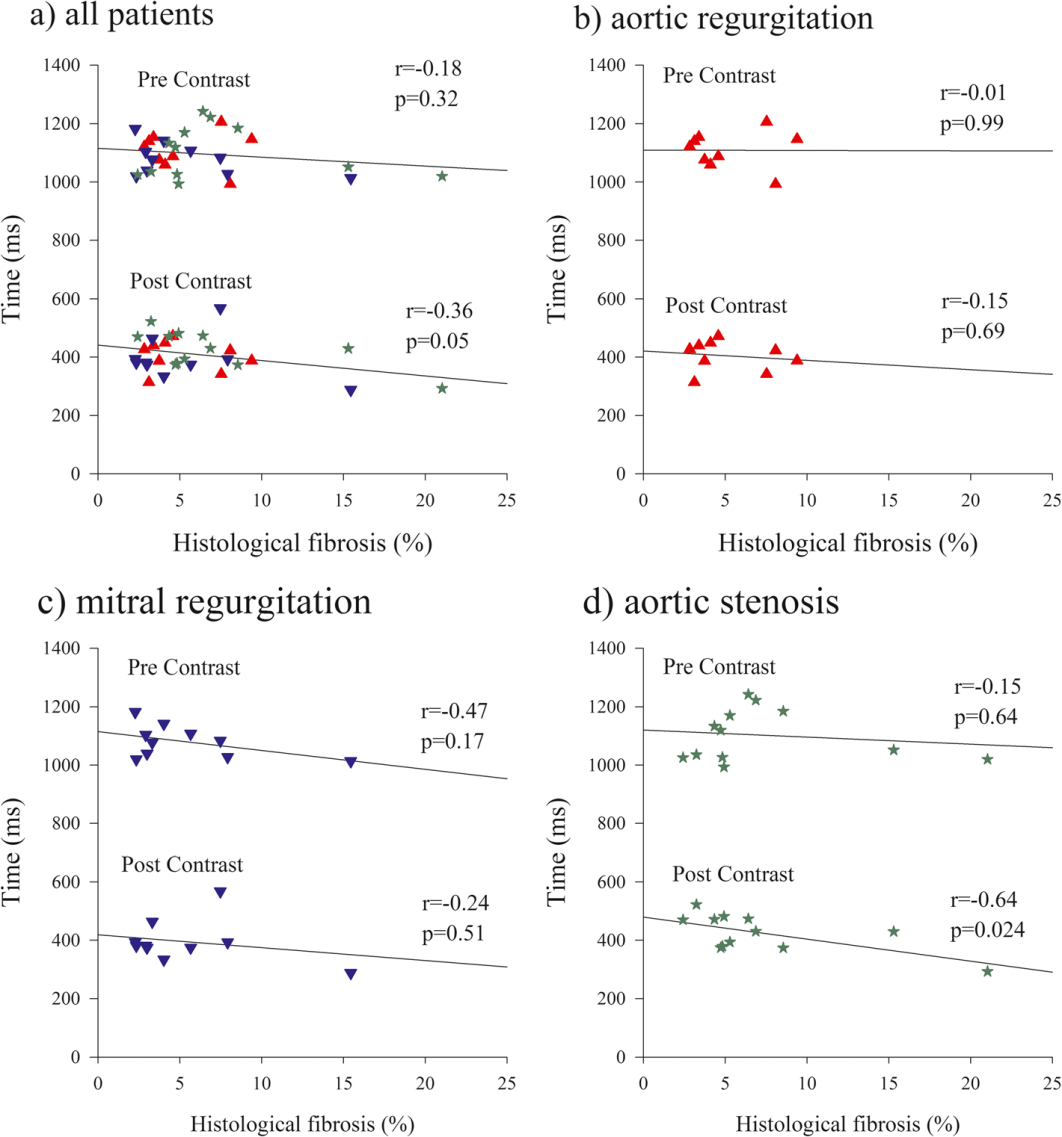
Correlation between pre and post contrast myocardial T1 times and fibrosis by histopathology. Correlation between pre and post-contrast myocardial T1 times and % histological fibrosis for the whole population panel (**a**) and individual curves in patients with aortic regurgitation panel (**b**) mitral regurgitation panel (**c**) and aortic stenosis panel (**d**)

**Fig. 4:**
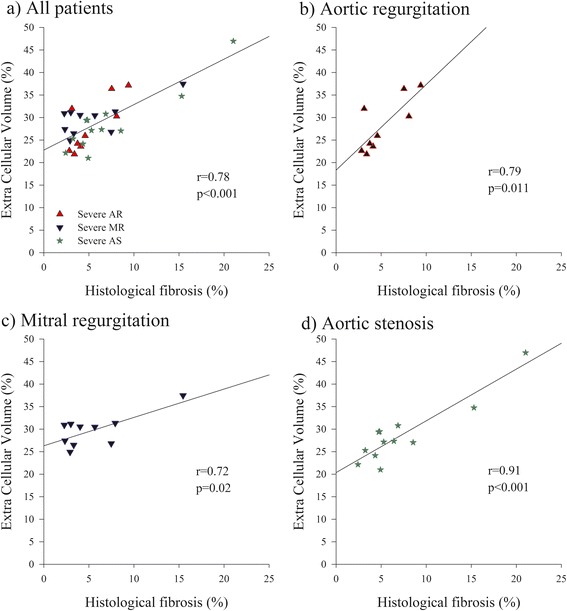
Correlation between ECV by Molli and fibrosis by histopathology. Correlation between ECV and % histological fibrosis for the whole population panel (**a**) and individual curves in patients with aortic regurgitation panel (**b**) mitral regurgitation panel (**c**) and aortic stenosis panel (**d**)

T1 times in blood and the myocardial segment corresponding to the biopsy site before and after contrast and computed ECV are shown in Table 2. Pre and post contrast T1 times did not differ among patients with different pathologies. There was no significant correlation between pre-contrast T1 time and the magnitude of histological fibrosis in either the whole population (r = −0.15, *p* = 0.41) or either of the three subgroup of patients with different pathologies (severe AR, [r = −0.01, *p* = 0.99], severe MR [r = −0.47, *p* = 0.17] and severe AS [r = −0.15, *p* = 0.64]). Post-contrast T1 time presented a significant but moderate inverse correlation with the amount of histological fibrosis in the entire group of patients (r = −0.36, *p* = 0.050). A significant correlation was only present in the subgroup of patients with severe AS (r = −0.64, *p* = 0.024, Fig. 3) but not in patients with severe AR (r = −0.15, *p* = 0.69) or severe MR (r = −0.24, *p* = 0.51). The average partition coefficient was 48.9 ± 8.6 % (range 34.4-79.6 %) and did not significantly vary among patients with different pathologies (aortic stenosis: 48.4 ± 11.0 %, aortic regurgitation: 47.7 ± 8.5 %, mitral regurgitation 50.7 ± 5.4 %, *p* = 0.73). The regional partition coefficient correlated well with the percent fibrosis by histopathology both in the whole group of patients (r = 0.68, p < 0.001) as well as in each of the  3 subgroup of patients with different valve diseases: ie in severe AR, (r = 0.69, *p* = 0.04), in severe MR (r = 0.58, *p* = 0.07) and in severe AS (r = 0.81, p < 0.001). E*CV derived from changes of post- to pre contrast myocardial and blood T1 times was 28.9 ± 5.5 % (range 21.0-47 %) and did not differ among patients with different pathologies (aortic stenosis: 30.1 ± 6.1 %, aortic regurgitation: 30.1 ± 5.1 %, mitral regurgitation 29.8 ± 3.4 %, *p* = 0.98). ECV was found to correlate strongly with histologically measured fibrosis both in the whole group of patients (r = 0.78, p < 0.001, Fig. 4). as well as in each of the 3 subgroup of patients with different valve diseases: ie in severe AR, (r = 0.79, *p* = 0.011), in severe MR (r = 0.72, *p* = 0.020) and in severe AS (r = 0.91, p < 0.001).

**Table 2:** Blood and myocardial T1 times and ECV in the region corresponding to the bioposy in different patient groups

	All patients	Aortic stenosis	Aortic regurgitation	Mitral regurgitation
	(*n* = 31)	(*n* = 12)	(*n* = 9)	(*n* = 10)
T1 Blood Pre-c. (ms)	1712 ± 99	1742 ± 131	1677 ± 59	1709 ± 79
T1 Myocardium Pre-c. (ms)	1097 ± 70	1103 ± 87	1109 ± 63	1080 ± 56
T1 Blood Post-c. (ms)	268 ± 47	277 ± 45	256 ± 34	268 ± 59
T1 Myocardium Post-c. (ms)	409 ± 64	425 ± 64	405 ± 52	395 ± 75
Hct (%)	40.9 ± 3.6	40.4 ± 4.9	41.1 ± 3.6	41.4 ± 1.6
λ (%)	48.9 ± 8.6	48.4 ± 11.0	47.7±8.5	50.7 ± 5.4
ECV (%)	28.9 ± 5.5	28.8 ± 6.9	28.2 ± 5.9	29.8 ± 3.6

Pre-c.:pre-contrast, Post-c.: post-contrast, Hc: Hematrocrit, ECV: Extra Cellular Volume, λ: partition coefficient

### Regional Varariation of myocardial T1 times, partition coefficient and ECV and its influence on correlation with histopathology

Segmental variation of myocardial T1 times before and after contrast and computed partition coefficient and ECV is shown in Table 3. Pre-contrast T1 times were significantly lower in the anterior segment than in other segments. However, post-contrast T1 times, computed partition coefficient and ECG did not significantly vary among segments. Correlations of ECV with histopathological fibrosis were similarly good when measurement were performed in anterior (r = 0.62, p < 0.001), anterolateral (r = 0.64, p < 0.001) inferoseptal (r = 0.70, P < 0.001) and anteroseptal (r = 0.64, p < 0.001) segments, or when ECV was computed from global T1 times of the whole LV slice (r = 0.74, p < 0.001) Correlations with histopathology were however poor / non existant for ECV measurements performed in inferolateral (r = 0.10, *p* = .35) and inferior (r = 0.22, *p* = 0.13) segments, likely because these 2 segments were more often affected by susceptibility artefacts on the  T1 maps.

**Table 3:** Segmental variations of pre and post contrast T1 times, partition coefficient and ECV on the mid-ventricular slice

	Entire LV	Anterior	Anterolateral	Infero-lateral	Inferior	Infero-septal	Antero-septal	P for segmental difference (Anova)
Pre contrast T1 time (ms)	1104 ± 59	1037 ± 108*	1111 ± 73	1117 ± 80	1136 ± 84	1131 ± 89	1094 ± 95	< 0.001
Post contrast T1 time (ms)	415 ± 59	412 ± 77	410 ± 64	427 ± 69	423 ± 61	417 ± 75	409 ± 68	0.73 (NS)
Partition coefficient λ (%)	48 ± 7	48 ± 12	50 ± 11	47 ± 10	47 ± 9	50 ± 22	49 ± 9	0.51 (NS)
ECV (%)	28 ± 4	29 ± 8	29 ± 6	28 ± 6	28 ± 5	30 ± 13	29 ± 5	0.54 (NS)

Pre-c.:pre-contrast, Post-c.: post-contrast, Hc: Hematrocrit, ECV: Extra Cellular Volume, λ: partition coefficient

### Reproducibility of measurements

**Fig. 5:**
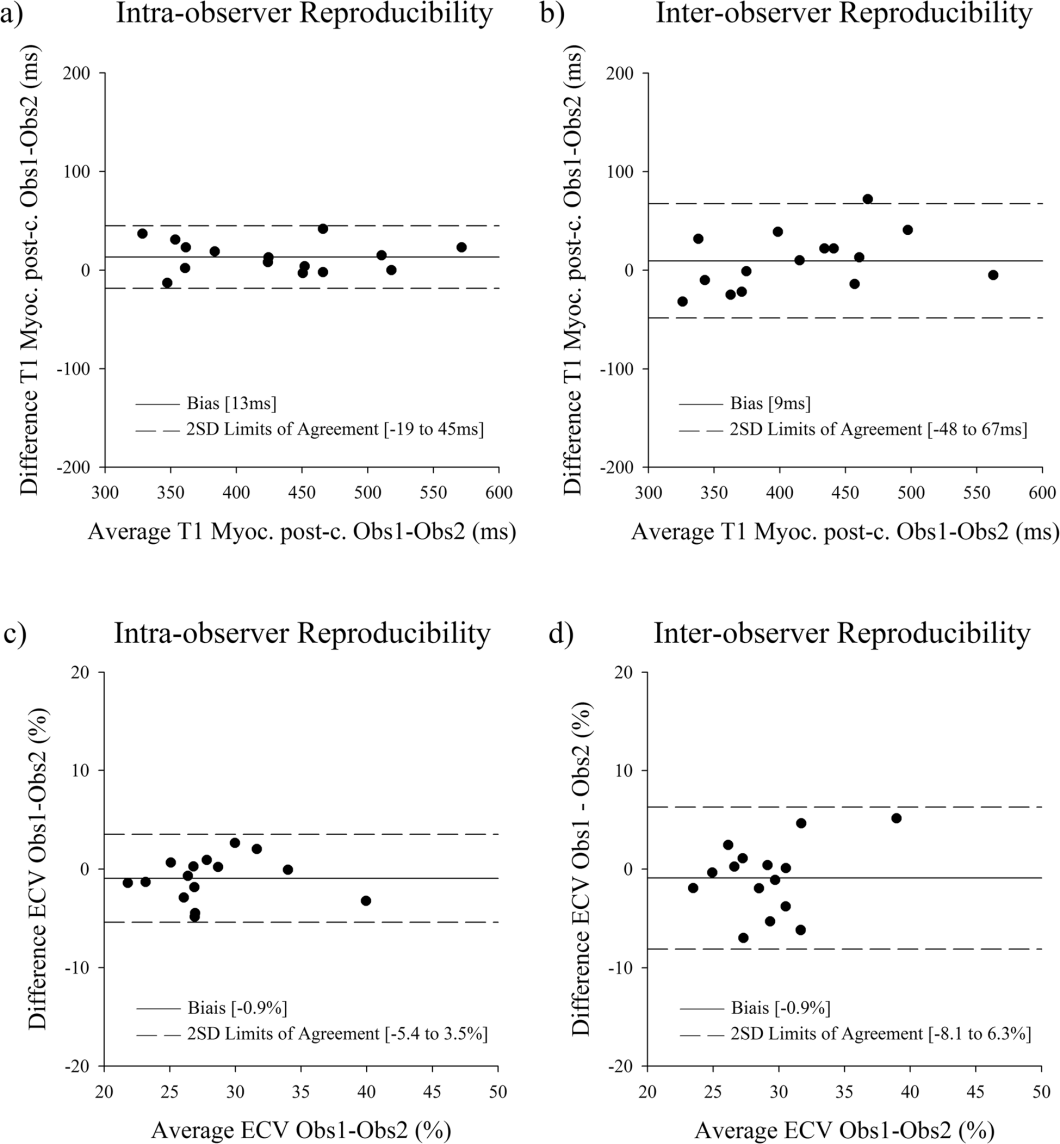
Bland-Altmann analysis of inter and intra observer reproducibility of T1 times and ECV. **a** intra observer and **b** inter-observer reproducibility of post-contrastT1 times measurement, **c** intra- and **d** inter-observer reproducibility of ECV measurements. Obs: Observer, Myoc: Myocardium, post-c.: post-contrast

Histological measurements of fibrosis had an intraobserver reproducibility (ICC) of 0.99 with a bias of 0 ± 2 % and interobserver reproducibility (ICC) of 0.82 with a bias of 0 ± 4 %. Intra and interobserver reproducibility for myocardial pre-contrast T1 times were ICC = 0.99 and ICC = 0.91 with bias of 7 ± 46 ms and 0 ± 55 ms respectively. Intra and interobserver reproducibility for post contrast T1 time measurement were ICC = 0.60 and 0.75 with bias of 13 ± 16 and 9 ± 29 ms respectively. Finally, intra- and inter-observer reproducibility for ECV were ICC = 0.51 and 0.61 with bias of −0.9 ± 2.2 % and −0.9 ± 3.6 % respectively (Fig. 5). Scan-rescan reproducibility of myocardial T1 time was ICC = 0.84 with a bias of 2 ± 39 ms. Scan-rescan reproducibility of ECV was ICC = 0.82 with a biais of −0.46 ± 2.4 %.

## Discussion

The salient findings of our study were:

1. ECV derived from 3 T CMR MOLLI T1 mapping, 15 min after bolus injection of Gd correlated well with the magnitude of diffuse interstitial myocardial fibrosis measured by picosirius red staining in histopathological samples of patients undergoing valve surgery.
2. By contrast, neither myocardial LGE extent nor non-contrast-enhanced T1 mapping of the myocardium were related to histologically measured fibrosis content.

There is currently intense interest in the detection of diffuse myocardial fibrosis by CMR, and several CMR techniques have been proposed for that purpose [14]. Most approaches make use of the unique fact that Gd based contrast agents have extravascular distribution volume, and shorten T1 time in direct proportion to their concentration. When myocardial tissue is replaced by focal replacement fibrosis, or when extracellular matrix proliferates due to diffuse interstitial fibrosis, the distribution volume of Gd-contrast agent in the heart increases. This leads to higher Gd concentration and shorter T1 times in areas of myocardial fibrosis, relative to normal tissue, when imaging is performed at time when the contrast agent has equilibrated between with blood (ie at equilibrium or several minutes after bolus injection). Accordingly it was proposed that the magnitude of interstitial myocardial fibrosis can be estimated by quantitative measurement of myocardial T1 times after contrast injection. Two approaches have been proposed: One makes use from computation of Gd-distribution volume after adjustment for contrast agent dose, relaxivity, postcontrast delay time, heart rate, and renal function [15]. The other approach relies on computation of ECV as the ratio of change in T1 times before and after contrast in myocardium relative to blood, corrected for haematocrit, once an equilibrium between plasma and myocardial contrast agent concentration has been reached [3, 16, 17]. Yet so far only few studies validated these approaches against histopathology [3–7, 18] and presently, all validation studies were performed only for 1.5 T. Also there have been only few reports on cardiac T1 mapping [10] and quantification of ECV at 3 T [19–23]. Indeed 3 T imaging may have some potential limitations as opposed to 1.5 CMR. It suffers from more pronounced inhomogeneities of both static magnetic field (B0) and the transmit radiofrequency field (B1), resulting in greater inhomogeneity of inversion and readout pulses and more pronounced off-resonance effects. Also pre-contrast times are longer in 3 T than 1.5 T, and may not fully recover when the same sampling schemes are used as for 1.5 T. This all might theoretically result in differences in estimates of ECV at 3 vs 1.5 T. Our present study is the first to demonstrate the accuracy of MOLLI T1 derived ECV at 3 T against histopathology. It confirmed excellent correlation for ECV measurements at 3 T against picrosirius red staining in septal biopsy samples, thereby validating 3 T measurements of ECV for estimates of diffuse intramyocardial fibrosis. This is in agreement with the work performed at 1.5 T [3–7, 18] and with other work [21, 22] demonstrating that field strength does not affect precision of T1 measurements and ECV, and that measurement of T1 had better scan/re-scan reproducibility at 3 T than 1.5 T.

Several recent reports suggested that T1 times before contrast are altered by interstitial fibrosis and that native non contrast enhanded-T1 mapping might be sufficient to detect fibrosis [8–10]. Such native T1 imaging not requiring contrast injection would have the advantage of being faster and easier than pre- post contrast ECV estimates. Also it would allow assessment of myocardial fibrosis in patients with contraindications to contrast, in particular in patients with renal failure. Therefore we also addressed this hypothesis in our study. In opposition to other work [10] we did not find any significant correlation between pre-contrast T1 times and histologically measured fibrosis in our study, questioning the feasibility of native contrast T1 imaging for the assessment of myocardial fibrosis at 3 T.

Finally we also compared LGE to histological fibrosis. Our present study employed an automated analysis method for quantification of LGE with high reproducibility, which was validated in animals against histology [13]. Consistent with our [11] and other [24–27] prior works, small areas of LGE in focal and patchy non-ischemic patterns were present in approximately 30 % of patients with severe valve disease. Yet, in contradiction to other studies [26–28], we did not observe a significant correlation between the extent of LGE and histologically measured fibrosis in this study. This is because LGE is mainly a measurement of focal replacement fibrosis rather than of diffuse interstitial fibrosis, and can only reveal areas of fibrosis which are at least the size of an imaging voxel. Thus the overall amount of myocardial fibrosis needs to be high to be revealed by LGE. Since biopsies were not performed exactly at the same level of LGE, it is also possible that the degree of focal replacement fibrosis fluctuates throughout the heart, and may be missed by biopsy samples from non-LGE areas. 

## Clinical implications

Several studies demonstrated usefulness of ECV for characterization of myocardial diseases, such as aortic valve disease, cardiomyopathies, hypertrophic cardiomyopathies and infiltrative diseases [1, 29]. Also myocardial post-contrast T1 times and ECV values have been associated with greater mortality [30, 31]. This present study confirms the accuracy of CMR estimates of ECV for detection of diffuse interstitial myocardial fibrosis at 3 T, suggesting that 3 T CMR can equally well be used for clinical estimates and risk stratification of patients with various cardiac diseases.

## Study limitations

Although biopsies and MOLLI images were sampled roughly the same region (the anterior or anteroseptal wall) of the heart, it is impossible to assure exact spatial correspondence between histopathology and CMR imaging. Also the biopsies samples are comparatively several thousand times smaller than the regions of interest evaluated by CMR and fibrosis in these small areas may not necessarily be representative of overall myocardial fibrosis, if fibrosis is not homogenously distributed through the heart which may be the case in diseased hearts. It has been recognized that T1 values can vary in different regions of the heart [32]. In our study, pre-contrast T1 was lower in anterior region than other segments, however post-contrast T1 values and ECV did not vary among segments. Also correlations of ECV with histopathology were similarly good for all segments, with the exception of inferior and inferolateral segments, probably because of susceptibility artefacts in these regions. Another limitation of our study was that only one single slice of MOLLI images was acquired. It was recently suggested that MOLLI images should be acquired on several slices to improve the accuracy of measurement. Also, to avoid overestimation of fibrosis in histopathological samples crossed by large blood vessels, we excluded perivascular fibrosis although it also is a part of extracellular volume. The picrosirius red staining used in our study is specific for collagen. Deposition of other extracellular proteins than collagen, for instance amyloid, can also increase ECV. Finally, ECV measured by CMR also includes vascular volume. Therefore changes in capillary blood volume might also theoretically affect the relation between ECV and interstitial fibrosis. Also the inclusion of vascular volume and of proteins other than collagen likely also explains why ECV is higher than collagen content, and why the regression plot between ECV and fibrosis in Fig. 4 estimates that ECV in patients without fibrosis would be approximately 23 %. This is however consistent with ECV values measured by other tracers such as ^14^C inulin [33, 34] ^14^C mannitol [34], Co-EDTA or TmDOTP^5−^ [35]. Finally it was recently suggested that ECV measurement may depend on contrast dose, since ECV  measured in healthy volunteers using high contrast doses (0.2 mmol/kg gadovist, as used in this work) were lower than for lower doses [31]. Whether this may have influenced the accuracy of our results remains however unknown.

## Conclusions

Our results indicate that ECV determined by 3 T CMR T1 MOLLI images closely correlates with histologically determined diffuse interstitial fibrosis, demonstrating the accuracy of this method for non-invasive quantification of interstitial fibrosis in patients with myocardial diseases. By opposition, non-contrast T1 mapping and LGE were not found to represent reliable measurements of interstitial fibrosis in patients with valve disease at 3 T.
